# Supplementary material for: A Proof of Principle Proteomic Study Detects Dystrophin in Human Plasma: Implications in DMD Diagnosis and Clinical Monitoring
Source: Int J Mol Sci. 2023 Mar 8;24(6):5215. doi: 10.3390/ijms24065215 (PMC10049465; doi:10.3390/ijms24065215)
Supplement: Supplementary file 1 [file ijms-24-05215-s001.zip › Supplementary Figure S1.pdf]

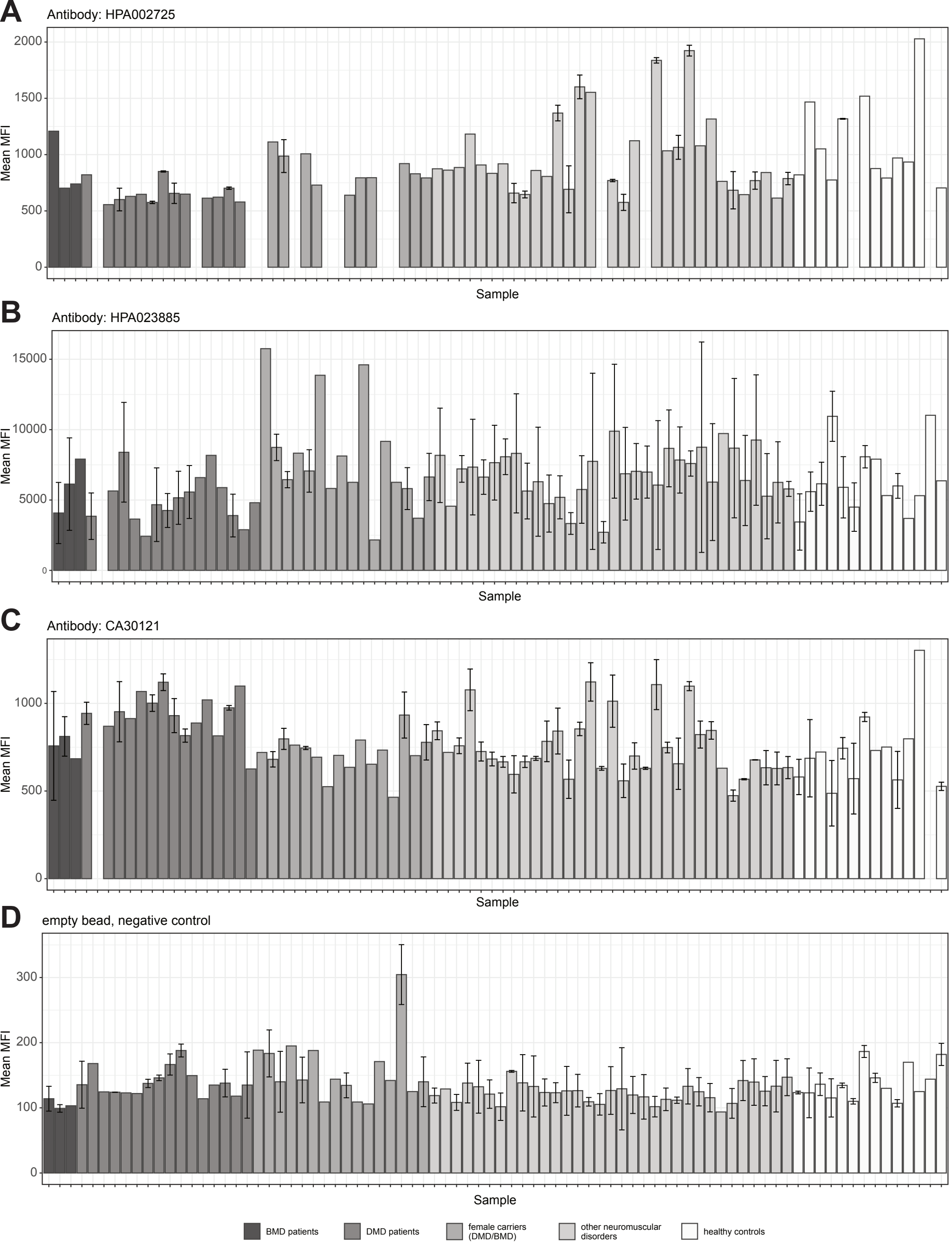
A
Antibody: HPA002725
Mean MFI
Sample
B
Antibody: HPA023885
Mean MFI
Sample
C
Antibody: CA30121
Mean MFI
Sample
D
empty bead, negative control
Mean MFI
Sample
BMD patients
DMD patients
female carriers (DMD/BMD)
other neuromuscular disorders
healthy controls
