# Supplementary material for: A Proof of Principle Proteomic Study Detects Dystrophin in Human Plasma: Implications in DMD Diagnosis and Clinical Monitoring
Source: Int J Mol Sci. 2023 Mar 8;24(6):5215. doi: 10.3390/ijms24065215 (PMC10049465; doi:10.3390/ijms24065215)
Supplement: Supplementary file 1 [file ijms-24-05215-s001.zip › Supplementary Table S1.pdf]

Supplementary Table S1

Peptides	Part of Dystrophin	Retention time
YQSEFEEIEGR	D1	4.1
LSSQLVEHCQK	D1	2.32
IQNHIQTLK	D1	2.38
WMAEVDVFLK	D1	8.6
EEWPALGDSEILK	D1	6.96
LLVSDIQTIQPSLNSVNEGGQK	D1	6.6
ELETLTTNYQWLCTR	D2	6.88
TLEEVWACWHELLSYLEK	D2	13.64
WLNEVEFK	D2	5.55
TTENIPGGAEEISEVLDSLENLMR	D2	13.65
HSEDNPNQIR	D2	0.83
ELHEEAVR	D2	1.4
LLEQSIQSAQETEK	D2	3.54
SLHLIQESLTFIDK	D2	8.32
QLAAYIADK	D2	3.75
VDAAQMPQEAQK	D2	2.41
IQSDLTSHEISLEEMK	D2	5.11
