# Supplementary material for: A Proof of Principle Proteomic Study Detects Dystrophin in Human Plasma: Implications in DMD Diagnosis and Clinical Monitoring
Source: Int J Mol Sci. 2023 Mar 8;24(6):5215. doi: 10.3390/ijms24065215 (PMC10049465; doi:10.3390/ijms24065215)
Supplement: Supplementary file 1 [file ijms-24-05215-s001.zip › Supplementary Table S2.pdf]

Supplementary Table S2

Peptides	Precursor (m/z)	Quantifier	Qualifier	Quantifier m/z	Qualifier m/z
YQSEFEEIEGR	693.8122^{+2}	b2+	y6+	292.1292	732.3523
LLVSDIQTIQPSLNSVNEGGQK	780.7517^{+3}	b2+	y12++	227.1754	615.3097
TTENIPGGAEEISEVLDSLENLMR	873.0936^{+3}	b4+	y4+	446.1882	533.2864
