# Supplementary material for: A Proof of Principle Proteomic Study Detects Dystrophin in Human Plasma: Implications in DMD Diagnosis and Clinical Monitoring
Source: Int J Mol Sci. 2023 Mar 8;24(6):5215. doi: 10.3390/ijms24065215 (PMC10049465; doi:10.3390/ijms24065215)
Supplement: Supplementary file 1 [file ijms-24-05215-s001.zip › Supplementary Table S3.pdf]

Supplementary Table S3

Number	Gender	Age (years)	Diagnosis	Group
1	M	4	BMD	BMD patients
2	M	34	BMD	BMD patients
3	M	59	BMD	BMD patients
1	M	6	DMD	DMD patients
2	M	9	DMD	DMD patients
3	M	8	DMD	DMD patients
4	M	5	DMD	DMD patients
5	M	6	DMD	DMD patients
6	M	6	DMD	DMD patients
7	M	8	DMD	DMD patients
8	M	6	DMD	DMD patients
9	M	6	DMD	DMD patients
10	M	7	DMD	DMD patients
11	M	5	DMD	DMD patients
12	M	5	DMD	DMD patients
13	M	6	DMD	DMD patients
14	M	6	DMD	DMD patients
15	M	11	DMD	DMD patients
16	M	14	DMD	DMD patients
1	M	65	HC	healthy controls
2	M	37	HC	healthy controls
3	M	64	HC	healthy controls
4	M	74	HC	healthy controls
5	M	21	HC	healthy controls
6	M	17	HC	healthy controls
7	M	45	HC	healthy controls
8	M	78	HC	healthy controls
9	M	83	HC	healthy controls
10	M	33	HC	healthy controls
11	M	36	HC	healthy controls

12	M	20	HC	healthy controls
13	M	18	HC	healthy controls
14	M	34	HC	healthy controls
1	M	45	Peripheral Neuropathy	other neuromuscular disorders
2	M	41	Peripheral Neuropathy	other neuromuscular disorders
3	M	17	Episodic ataxia type 2	other neuromuscular disorders
4	M	15	Possible Thomsen/Becker	other neuromuscular disorders
5	M	37	Collagenopaty	other neuromuscular disorders
6	M	51	Bethlem myopathy	other neuromuscular disorders
7	M	72	Myotonic Dystrophy Type 1	other neuromuscular disorders
8	M	55	Congenital myasthenia	other neuromuscular disorders
9	M	21	Atrial Arrythmias	other neuromuscular disorders
10	M	22	Myofibrillar myopathy	other neuromuscular disorders
11	M	46	Ataxia	other neuromuscular disorders
12	M	63	Ataxia, spasticity	other neuromuscular disorders
13	M	65	Huntington Disease	other neuromuscular disorders

14	M	61	Huntington Disease	other neuromuscular disorders
15	M	47	Huntington Disease	other neuromuscular disorders
16	M	63	Huntington Disease	other neuromuscular disorders
17	M	67	Huntington Disease	other neuromuscular disorders
18	M	66	Myopathy	other neuromuscular disorders
19	M	77	Neuropathy	other neuromuscular disorders
20	M	65	Neuropathy	other neuromuscular disorders
21	M	53	Nemaline myopathy	other neuromuscular disorders
22	M	38	Parkinson disease	other neuromuscular disorders
23	M	54	Motor and sensory Neuropathy	other neuromuscular disorders
24	M	35	Charcot-Marie-Tooth disease type 1A	other neuromuscular disorders
25	M	50	Hereditary neuropathy	other neuromuscular disorders
26	M	58	Peripheral neuropathy	other neuromuscular disorders
27	M	66	Dystonia 16	other neuromuscular disorders
28	M	53	Ataxia	other neuromuscular disorders

29	M	34	LGMD 2D	other neuromuscular disorders
30	M	44	spinal muscular atrophy type 3	other neuromuscular disorders
31	M	50	Spastic ataxia	other neuromuscular disorders
32	M	44	Spastic ataxia	other neuromuscular disorders
33	M	47	Thomsen Myotonia	other neuromuscular disorders
1	F	41	DMD familial history	female carriers
2	F	43	DMD familial history	female carriers
3	F	38	DMD familial history	female carriers
4	F	18	DMD familial history	female carriers
5	F	40	DMD family history	female carriers
6	F	68	BMD family history	female carriers
7	F	32	BMD family history	female carriers
8	F	42	DMD family history	female carriers
9	F	31	DMD family history	female carriers
10	F	43	DMD familiar history	female carriers
11	F	40	BMD family history	female carriers
12	F	49	DMD family history	female carriers
13	F	45	DMD family history	female carriers
14	F	37	DMD family history	female carriers
